# Integrative Machine Learning of Genetic and Lifestyle Factors for Personalized Skin Health

**DOI:** 10.1109/JTEHM.2026.3675676

**Published:** 2026-03-19

**Authors:** Yassine Benachour, Lina Maloukh, Barbara Geusens

**Affiliations:** Engineering Technology and ScienceHigher Colleges of Technology 62721 Dubai United Arab Emirates; College of Natural and Health SciencesZayed University 54483 Dubai United Arab Emirates; Nomige 9000 Ghent Belgium

**Keywords:** Dermatogenomics, multimodal data integration, gene–environment interactions, k-modes clustering, nested cross-validation, leakage-free evaluation, XGBoost, model interpretability, permutation importance, SHAP

## Abstract

Objective: To develop an AI framework that combines genetic, phenotypic, and lifestyle data for profiling skin-health patterns and generating hypothesis-supporting summaries for potential decision support.Methods and procedures: A dataset of 5,254 individuals integrates six genes (FLG, AQP3, MMP-1, MMP-3, SOD2, GPX), six phenotype severities, and 20+ lifestyle factors. Mutation burden and interactions are tested by ANOVA. K-modes clustering identifies four interpretable dermatological profiles within the cohort and is embedded in leakage-free nested cross-validation (train-only selection; test labels from training centroids). Subtypes are predicted from genetics plus lifestyle using an XGBoost (XGB) classifier; explainability uses gain, permutation importance, and SHAP contributions aggregated across outer folds.Results: Four subtypes are identified. Mutation burden differed across phenotypes (ANOVA, $p< 0.05$). Interactions are observed for AQP3 $\times $Winter $\rightarrow $Dryness, GPX $\times $Medication $\rightarrow $Pigmentation, and MMP-3 $\times $City Living $\rightarrow $Redness. Nested-CV prediction achieves $0.9789\pm 0.0083$ accuracy with macro-F $1~0.9711\pm 0.0126$ and macro-recall $0.9697\pm 0.0091$. This outperformed unimodal baselines and improved generalization across all folds in practice. Drivers are stable across folds and included scrub usage, stress, sleep, low water intake, menopause, and camouflage habits, alongside oxidative-stress and MMP genes.Conclusion: Integrating genomic susceptibility with modifiable exposures enables robust, interpretable skin-profile prediction and highlights actionable targets for stratified counseling beyond genetic predisposition.

## I. Introduction

Skinhealth reflects a complex interplay between genetic predisposition and lifestyle exposures. Variants in genes such as FLG, AQP3, MMP-1, MMP-3, SOD2, and GPX are known to influence barrier integrity, hydration, oxidative stress response, and dermal remodeling. For example, mutations in FLG are associated with atopic dermatitis due to impaired skin barrier function [1], [2], [3], while *AQP3* regulates epidermal hydration via water and glycerol transport [4], [5]. *SOD2* and *GPX* play key roles in antioxidant defense [6], and *MMP-1*/*-3* facilitate collagen turnover, particularly under UV exposure [7], [8].

However, genetic predisposition alone does not fully determine dermatological outcomes. Environmental and behavioral factors—such as UV radiation, pollution, stress, exfoliation, and smoking—interact with genetic background to modulate skin phenotypes [9], [10], [11]. Excessive sun exposure, for instance, may accelerate pigmentation and collagen degradation even in genetically low-risk individuals [12], [13], while smoking or abrasive exfoliation can worsen acne or sensitivity, particularly in those with compromised antioxidant or inflammatory pathways [14]. These findings support the need for integrative approaches that consider both intrinsic and extrinsic drivers of skin health.

Recent advances in artificial intelligence (AI) and machine learning (ML) enable scalable modeling of such gene–environment interactions. Deep learning has demonstrated high accuracy in dermatological imaging tasks, including lesion classification and skin cancer detection [15], [16], [17], [18]. Liu et al. [19] developed an AI-based system that leverages consumer genetic profiles for skincare recommendations, highlighting the potential of genomics-guided personalization. Yet, most current models rely on image-based diagnostics and underutilize individual-level genetic and lifestyle data [20], [21], [22]. Integrating multi-modal information remains an emerging frontier in dermatogenomics and precision care. A consolidated list of abbreviations and acronyms is provided in *Supplementary 9*.

The convergence of digital health, behavior tracking, and genomics presents new opportunities for personalized dermatology [14], [23], [24], [25]. Studies increasingly emphasize the importance of integrating lifestyle variables—such as sun exposure, physical activity, and exfoliation—into predictive frameworks [26], aligning with the broader “lifestylopathy” paradigm in chronic disease management.

Despite progress, notable gaps persist. Personalized skincare solutions remain largely generalized, with limited use of genotypic or behavioral data. Genome-wide studies have identified disease-associated variants, but few link these insights to actionable skincare interventions. Moreover, ML applications in dermatology rarely account for dynamic lifestyle factors that influence disease progression [27], [28].

This study addresses these gaps by leveraging a real-world dataset of 5,254 individuals from a consumer skincare platform. The dataset includes genotypic information for six skin-relevant genes, over 20 lifestyle/environmental variables, and self-reported skin concern severities. We apply ensemble clustering and supervised learning to (1) identify dermatological subtypes, (2) evaluate genetic and lifestyle contributions to skin concern severity, and (3) build interpretable ML models for personalized skincare guidance.

Our work contributes a novel framework for integrative dermatogenomics by extending ensemble-based clustering techniques [20], [21] to real-world consumer health data. By modeling gene–lifestyle interactions and integrating multiple data modalities, we aim to support proactive and personalized skin health strategies at scale.

From a translational perspective, the proposed pipeline targets dermatology-oriented risk stratification and decision support using routinely collected genetic and lifestyle data. In practice, each individual is assigned an interpretable phenotype risk cluster, accompanied by the dominant contributing drivers (genetic markers and modifiable lifestyle exposures) and mapped intervention categories to guide personalized prevention and management, including barrier support, exposure mitigation, and routine adjustment. The end-to-end workflow from data acquisition to decision support output is summarized in Fig. 8.

**FIGURE 1.**
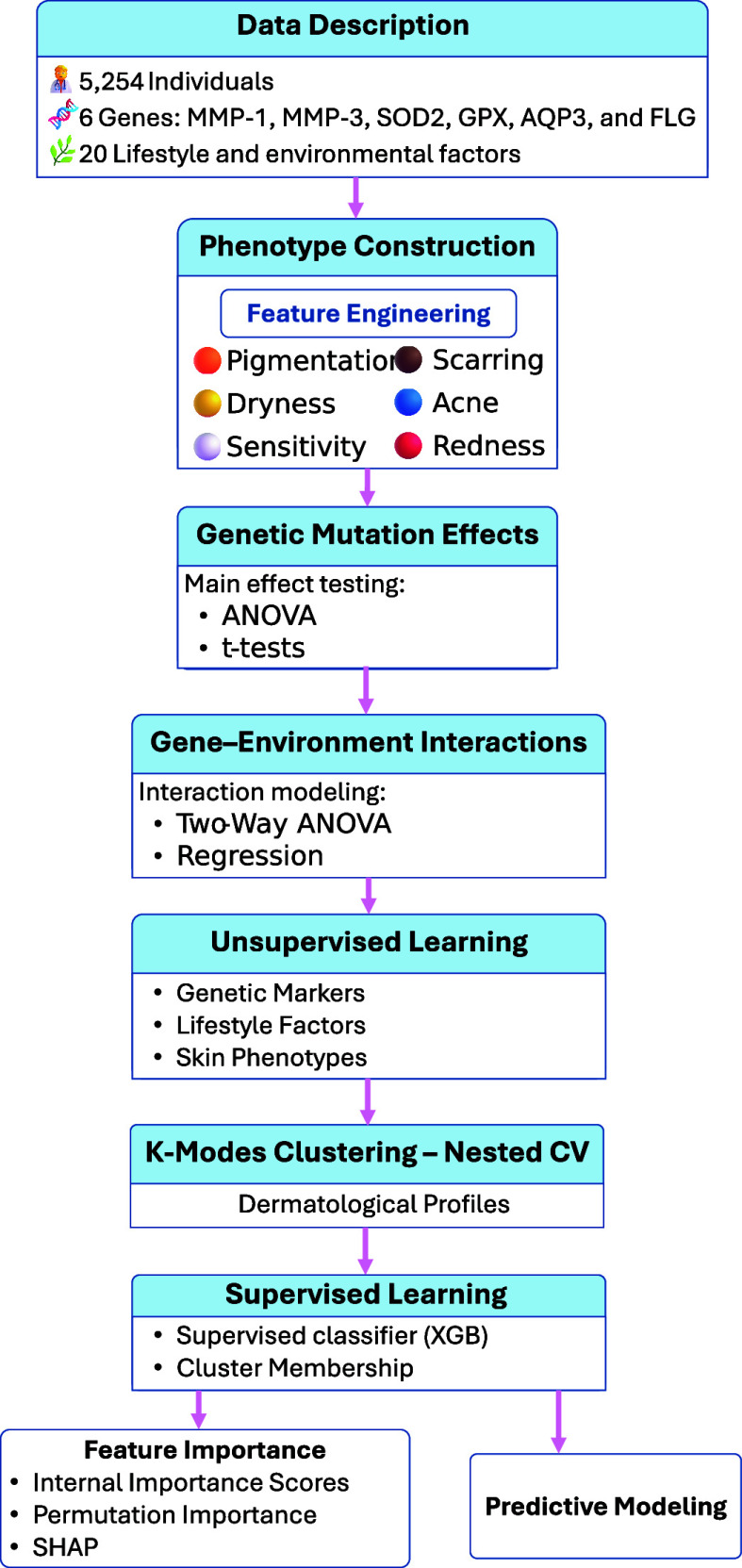
**Overall study workflow**. Data from 5,254 individuals include a six-gene panel, lifestyle factors, and engineered phenotypes. Statistical analyses assess genetic main effects and gene–environment interactions. K-modes identifies dermatological profile clusters. A supervised model predicts cluster membership, and feature importance supports interpretation.

The following section outlines our analytical objectives and hypotheses, which guide the development and validation of the proposed machine learning framework.

### A. Objectives and Hypotheses

The primary objective of this study is to evaluate how genetic predispositions and modifiable lifestyle factors collectively influence common skin concerns, and to develop an interpretable machine learning framework for personalized skincare. Specifically, we aim to (1) integrate genotypic data with lifestyle and environmental variables (e.g., diet, sun exposure, skincare routines), (2) develop and validate clustering and classification models for individualized skin-profile prediction, and (3) generate personalized recommendations by identifying high-risk genetic profiles along with their behavioral modifiers.

To support these goals, we test three hypotheses: (1) individuals with specific mutations in skin-related genes (e.g., *FLG*, *AQP3*, *MMP-1*, *MMP-3*, *SOD2*, *GPX*) exhibit greater severity in corresponding phenotypes such as dryness, redness, or sensitivity; (2) unfavorable lifestyle behaviors (e.g., sunbathing, exfoliation, smoking) exacerbate skin concerns in genetically predisposed individuals, indicating significant gene–environment interactions; and (3) predictive models that integrate both genetic and lifestyle features outperform those based on a single modality, demonstrating the added value of multi-modal integration for dermatological risk stratification.

Our framework provides a proof-of-concept for AI-driven dermatogenomic modeling, advancing precision skincare through integrated biological and behavioral insights.

## II. Methods

The methodological framework for this study integrates statistical testing, unsupervised learning, and predictive modeling to evaluate the role of genetic mutations, lifestyle behaviors, and their interactions in shaping dermatological phenotypes. An overview of the full pipeline is presented in Fig. 1. From a system-level perspective, the pipeline can be viewed as a lightweight decision-support workflow that operates on routinely obtainable consumer/clinic intake data. Inputs consist of (i) a lifestyle/environmental questionnaire (e.g., sun exposure, exfoliation practices, stress, hydration, smoking) and (ii) a targeted genomic panel for six skin-relevant genes (*MMP-1, MMP-3, SOD2, GPX, AQP3, FLG*). The model produces three output objects designed for downstream use: an assigned dermatological profile (phenotype risk cluster), a compact risk summary, and a ranked set of contributing drivers separating intrinsic (genetic) from modifiable (lifestyle) factors, which can be mapped to intervention categories (e.g., barrier support, exposure mitigation, routine adjustment). In a clinical or consumer-health setting, these outputs are intended to be reviewed by a dermatologist or delegated clinic staff (e.g., nurse/skin therapist) alongside the standard consultation, with deployment assumptions consistent with attachment to an electronic intake form or consumer portal and optional export into the electronic health record as a structured note or PDF report.

**FIGURE 2.**
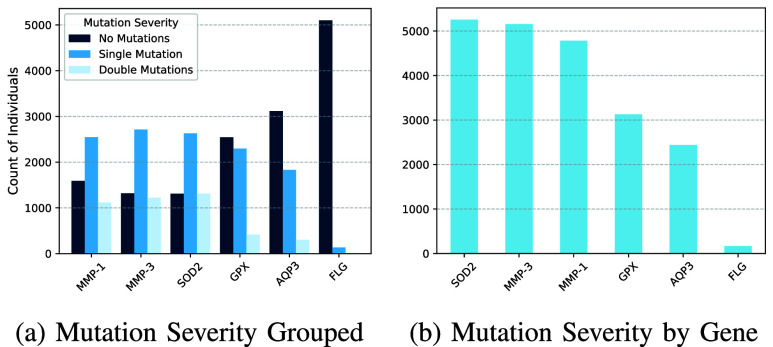
Comparison of mutation severity distributions.

The analysis begins with data description and phenotype construction, followed by statistical evaluation of the main effects of mutation burden and gene–environment interactions using ANOVA-based methods. These steps are then followed by k-modes clustering to derive dermatological subtypes from integrated multi-modal data. The resulting cluster labels serve as targets for supervised classification models (XGBoost), enabling prediction of skin profiles based on genetic and lifestyle inputs. Feature importance is assessed using both internal and permutation-based methods to identify the most informative predictors. The following subsections detail each component of this workflow.

### A. Dataset Description

This study utilizes a proprietary dataset provided by Nomige, a company specializing in DNA-based skincare personalization. The dataset comprises anonymized data from 5,254 individuals, integrating genetic, lifestyle, and phenotypic information to enable the development of machine learning models for personalized skin health prediction. Nomige’s approach bridges dermatogenetics and AI to inform skincare interventions. It leverages both inherent genetic predispositions and modifiable behavioral factors to personalize recommendations.

The dataset (Table 1) contains self-reported severity scores for six common skin phenotypes (pigmentation, dryness, sensitivity, scarring, acne, redness), alongside single nucleotide polymorphism (SNP)-based mutation profiles for six genes implicated in skin function: *MMP-1, MMP-3, SOD2, GPX, AQP3,* and *FLG*. Gene abbreviations follow standard nomenclature and are listed in full in *Supplementary 2*. These genes are functionally associated with collagen degradation (MMPs), oxidative stress response (*SOD2, GPX*), and barrier/hydration mechanisms (*AQP3, FLG*). Mutations are represented categorically as no mutation (0,0), single mutation (0,1 or 1,0), or double mutation (1,1).

**TABLE 1:** Dataset Description: Categories and Corresponding Columns

Category	Columns
Genetics	MMP-1, MMP-3, SOD2, GPX, AQP3, FLG
Phenotype	Pigmentation, Dryness, Sensitivity, Scarring, Acne, Redness
Lifestyle	20+ lifestyle/environmental variables

In addition to genetic markers, the dataset captures over 20 lifestyle and environmental factors on ordinal or binary scales—such as smoking status, sun exposure, exfoliation routines, stress levels, and hydration habits. Examples include *Camouflage Imperfections* (use of makeup to hide skin flaws), *Scrub.Usage* (use of granular exfoliants), and *Is_Winter* (seasonal indicator for cold/dry weather). See *Supplementary 1* for full variable definitions. We curated these variables to reflect modifiable behaviors that may interact with underlying genetic predispositions. Although participant age is not directly recorded, the dataset includes menopause and pregnancy status as proxies for life stage in female participants. Approximately 95% of participants are female (mostly ages 30–50), reflecting the product’s target demographic. This demographic bias should be considered when generalizing the results.

To explore genetic susceptibility patterns in the population, we conducted an initial mutation severity analysis across the six target genes. We define mutation severity categorically by the number of mutations (0, 1, or 2), where severity refers to the extent or level of genetic alterations in a particular gene, which quantifies the potential impact of mutations on gene function and associated biological traits. We visualized the results to compare the distribution of mutation states across genes, both in aggregate and stratified by severity levels.

Figure 2 displays the distribution of mutation severity (categorized as no mutations, single mutations, and double mutations) across the six genes under study. *FLG* exhibited the highest proportion of individuals with no mutations, suggesting robust genetic conservation in this barrier-associated gene in this cohort. In contrast, AQP3 had the fewest individuals without mutations, suggesting that its function in hydration may be more vulnerable to disruption in this cohort. Single mutations are the most common mutation category across all genes, especially for *MMP-1*, *MMP-3*, and *SOD2*, where heterozygous variants occurred frequently. Double mutations are relatively infrequent but are notably present in *MMP-1, MMP-3*, and *SOD2*, reflecting greater mutational diversity in these genes.

**FIGURE 3.**
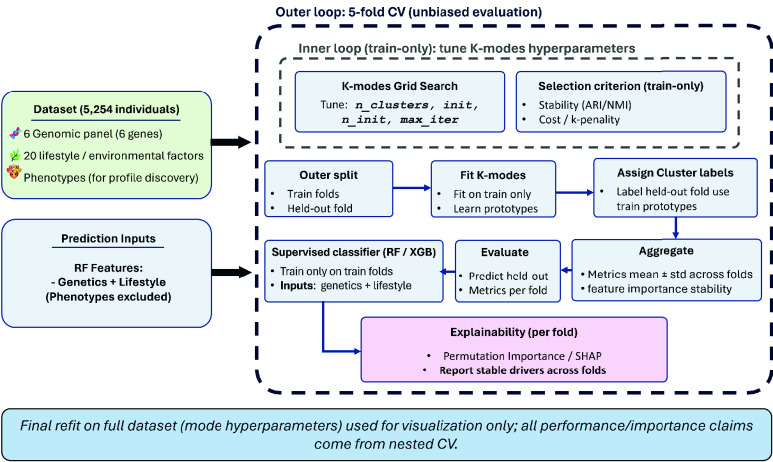
Leakage-free nested cross-validation pipeline for clustering, labeling, and prediction. An outer 5-fold loop yields unbiased evaluation. Within each outer-training split, an inner train-only procedure selects k-modes hyperparameters using stability and mismatch cost with a mild $k$-penalty. k-modes is fit on the outer-training data only and assigns labels to the held-out fold using training-derived prototypes. The classifier (XGBoost) is trained on genetics plus lifestyle and evaluated on the held-out fold. Metrics and explainability reported as mean ± std across outer folds.

When aggregating mutation severity across genes, SOD2 demonstrated the highest cumulative burden, followed closely by MMP-3 and MMP-1 (Fig. 2b). These genes, central to oxidative stress mitigation and extracellular matrix remodeling, may therefore represent high-priority biomarkers for skin aging and resilience. Meanwhile, GPX and AQP3 displayed moderate mutation loads, supporting their relevance in redox and hydration pathways. FLG, by contrast, maintained the lowest total mutation severity, reinforcing its evolutionary stability and critical role in skin barrier integrity. These observations provide a biologically meaningful foundation for subsequent clustering and predictive modeling efforts aimed at individual-level skin concern risk profiling.

All records were collected by the commercial provider under informed consumer consent and were provided to the authors in fully de-identified form; the analysis constituted retrospective use of anonymized data without participant contact or intervention and was determined to be exempt / not requiring institutional review under applicable guidance.

Next, we describe the feature engineering process applied to DNA, lifestyle, and phenotype data in order to enable interpretable and accurate skin health predictions.

### B. Data Preprocessing: Feature Engineering

Phenotypic variables are derived from open-ended responses to the intake survey question, “What are your skin concerns?” These textual responses are preprocessed and mapped to six standardized phenotype categories: *Pigmentation*, *Dryness*, *Sensitivity*, *Scarring*, *Acne*, and *Redness* (*see Supplementary 3*). Each response is then aligned with an ordinal severity level—ranging from no concern to severe—based on the presence and intensity of keywords identified through manual annotation. The encoding schema is designed to reflect logical severity progression, ensuring consistency across phenotypes and enabling downstream models to treat these as structured ordinal targets.

This phenotypic engineering step involved the extraction and transformation of qualitative responses through manual labeling and categorical mapping. Given the variability and ambiguity in the original free-text data, this harmonization process is essential for converting unstructured user input into high-quality structured variables. An overview of the full ordinal encoding framework is presented in *Supplementary 4*, where each phenotype follows a logically escalating severity scale. Notably, individuals could report multiple concerns simultaneously; thus, phenotypes are encoded independently, resulting in a multi-label structure that captures the presence and severity of each issue per respondent.

To support reproducibility and transparency, the rule-based encoding system used to map free-text responses to phenotype categories and severity levels is detailed in *Supplementary 5*. The diversity of expressions—for example, “tightness after shower,” “rash and flaky skin,” or “bags under the eyes”—required careful interpretation within a dermatological context. This necessitated iterative rule construction and validation in collaboration with domain experts. The final distribution of severity levels across phenotypes is illustrated in *Supplementary 6*.

Preprocessing also extended to genetic and behavioral data. Genotype parsing involved the development of custom scripts to separate and encode individual mutation states consistently across all records. Mutation burden for each gene is categorized as 0 (wild type), 1 (heterozygous), or 2 (homozygous or compound heterozygous), and this encoding scheme is applied uniformly across statistical and machine learning workflows.

Lifestyle and environmental variables—spanning over 20 dimensions—underwent normalization, deduplication, and standardization (e.g., mapping variables recorded on 0–1 or 0–3 scales). Ambiguous or inconsistent entries are flagged and resolved using logic-based harmonization rules. The result is a dataset composed entirely of categorical variables, encoded as ordinal or nominal depending on the nature of each feature. This uniform representation facilitated compatibility with various machine learning algorithms while preserving semantic integrity. Our emphasis on a structured feature-engineering workflow and importance-driven interpretability as part of model optimization follows an integrated pipeline perspective previously validated in a separate biomedical machine-learning setting [29].

Rigorous preprocessing is essential for ensuring that the dataset could support valid analytical outcomes. By structuring phenotypes into ordinal severity levels and harmonizing genetic and behavioral data, we enabled models to capture the nuanced, multi-factorial patterns of skin health.

The resulting dataset provides a robust multi-modal foundation for subsequent analyses. In the next section, we present statistical evaluations of the relationship between genetic burden and phenotype severity, including one-way ANOVA, post-hoc $t$-tests, and two-way ANOVA to explore gene–environment interactions. These analyses support the discovery of latent skin health profiles and inform downstream clustering and classification tasks.

### C. Statistical Analysis

#### 1) Inferential Analysis of Mutation Burden on Dermatological Traits

To evaluate **Hypothesis 1**, which investigates whether individuals carrying a greater burden of mutations in skin-related genes exhibit increased severity in corresponding phenotypes, we conducted a series of inferential statistical tests focusing on six dermatological concerns: Pigmentation, Dryness, Sensitivity, Scarring, Acne, and Redness. We encoded each phenotype on an ordinal severity scale and stratified participants by their mutation status for skin-related genes into three groups: no mutations, single mutation, and double mutations (homozygous or compound heterozygous).

We conducted one-way ANOVA to compare mean phenotype severities across the three mutation groups (no, single, double). Following the identification of significant group-level effects, we conducted post-hoc pairwise t-tests to identify specific group differences (i.e., no mutation vs. single mutation, no mutation vs. double mutation, and single mutation vs. double mutation). These t-tests allowed us to pinpoint where significant differences emerged in phenotype severity between mutation burden levels while controlling for within-group variance.

We performed all analyses independently for each skin phenotype and set statistical significance at $p < 0.05$. We conducted the analyses using Python’s scipy.stats and statsmodels libraries to ensure reproducibility and statistical transparency. While these results confirm that genetic mutations are significantly associated with differences in skin phenotype severity, they do not account for the potential influence of lifestyle and environmental factors. To explore whether these variables interact with genetic predispositions to modulate dermatological outcomes, we next conducted a series of gene–environment interaction analyses using two-way ANOVA.

#### 2) Modeling Gene–Environment Interactions Using Two-Way ANOVA

In addition to modeling the additive effects of genetic variants and lifestyle behaviors, we investigated the presence of biologically plausible gene–environment interactions to capture more complex, non-additive dynamics influencing skin phenotype severity. This step reflects the real-world complexity of skin health, where outcomes are often shaped by the synergy between inherited traits and modifiable environmental exposures.

We focused on five gene–lifestyle combinations based on established biological mechanisms, dermatological literature, and exploratory data analysis. Each hypothesis reflects a plausible scenario in which a genetic mutation could amplify or buffer the effect of a lifestyle or environmental factor. The five tested interactions are:

• **FLG** $\times $ **Scrub Usage** $\rightarrow $ **Sensitivity:** FLG mutations impair skin barrier integrity, increasing susceptibility to external irritation and atopic dermatitis [30], [31], [32]. We hypothesized that frequent mechanical exfoliation would exacerbate sensitivity in FLG mutation carriers.
• **MMP-** $3\times $ **City Living** $\rightarrow $ **Redness:** MMP genes regulate collagen degradation and inflammatory responses. Environmental pollutants like particulate matter can trigger inflammation and matrix remodeling, potentially intensifying redness in individuals with MMP mutations [33], [34], [35].
• **SOD** $2\times $ **Stress** $\rightarrow $ **Acne:** SOD2 encodes a key mitochondrial antioxidant enzyme. Reduced oxidative defense due to mutation, when combined with psychosocial stress, may lead to heightened inflammatory response and increased acne severity [8], [36].
• **AQP** $3\times $ **Winter Exposure** $\rightarrow $ **Dryness:** AQP3 facilitates trans-epidermal water transport. Cold, dry environments exacerbate moisture loss, particularly in individuals with impaired water channel function due to AQP3 mutations [5], [33], [37], [38].
• **GPX** $\times $ **Medications** $\rightarrow $ **Pigmentation:** GPX protects cells from oxidative damage. Photosensitizing or oxidative-stress-inducing medications (e.g., isotretinoin, hormonal treatments) may have amplified pigmentary effects in individuals with GPX variants [6], [8], [39].

To test these hypotheses, we used two-way ANOVA models to examine the main and interaction effects of genotype (mutated vs. non-mutated) and lifestyle factor (binary or binned continuous variable) on each skin phenotype. We binarized gene mutation status (0 = no mutation; 1 = one or more mutations) and either used lifestyle variables directly (if binary) or split them into “Low” and “High” categories using median or quantile-based binning. We assessed the interaction effect using type II sum-of-squares ANOVA to determine whether the impact of a lifestyle factor is significantly modulated by genetic status.

In addition to linear ANOVA, we also ran non-linear tree-based models to confirm and visualize the interaction patterns. We generated interaction plots for each hypothesis, displaying phenotype severity across environmental levels for carriers vs. non-carriers. This multi-method approach enhances both the interpretability and rigor of our findings and strengthens support for Hypothesis 2 by demonstrating that the genetic contribution to skin health cannot be fully understood in isolation from behavioral context.

Building on this foundation, the next stage of our analysis explores whether patterns of skin phenotypes, informed by both genetics and lifestyle variables, can be further stratified using unsupervised learning techniques. We now turn to clustering methods—specifically the application of k-modes—to uncover latent skin profiles within this multi-modal dataset.

### D. Clustering Analysis of Integrated Skin Profiles

To capture heterogeneity in integrated skin profiles arising from genetic variation and lifestyle factors, we combined unsupervised phenotyping with supervised validation under a strictly partitioned evaluation protocol. All variables are preprocessed as categorical or ordinal features, including encoded gene variants, discretized phenotype grades, and questionnaire-derived lifestyle/environmental attributes. Accordingly, we adopted k-modes clustering, which is designed for categorical data and assigns membership by minimizing mismatches to cluster modes.

Clustering, label assignment, and predictive evaluation are conducted within a leakage-free nested cross-validation framework (Fig. 3) so that cluster discovery and hyperparameter selection are confined to training data in each evaluation split. The outer 5-fold loop defines held-out folds for unbiased assessment. Within each outer-training split, k-modes hyperparameters are selected over a fixed candidate grid: $n_{\text {clusters}} \in \{3,4,5\}$, $\texttt {init} \in \{\texttt {Huang}, \texttt {Cao}\}$, $n_{\text {init}} \in \{5,10,20\}$, and $\mathtt {max\_iter} \in \{100,300,500\}$.

**FIGURE 4.**
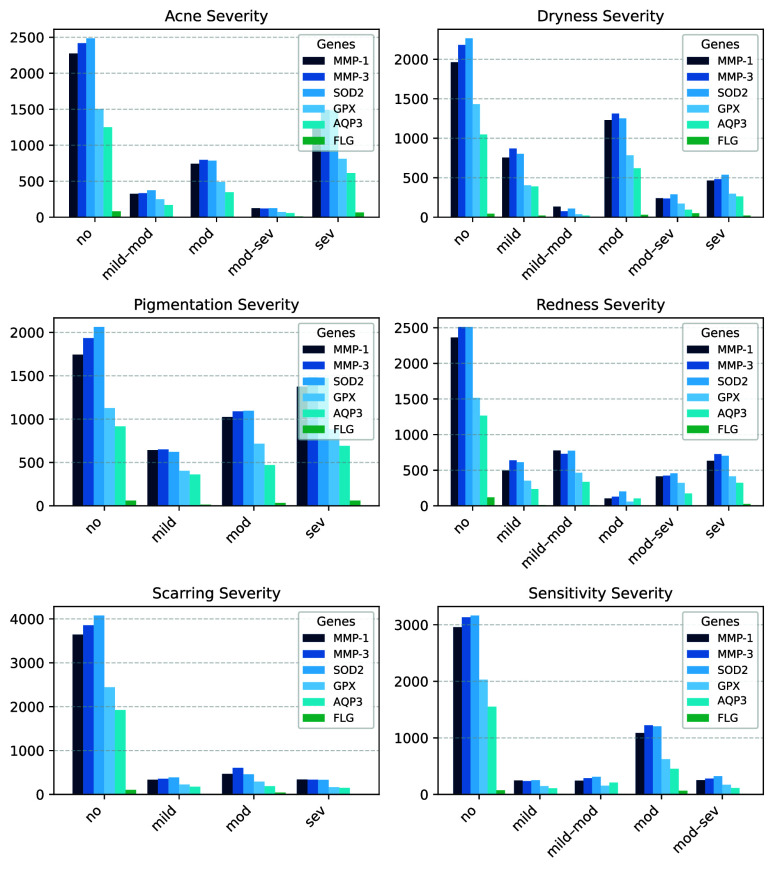
Sum of mutation counts per gene across severity levels for each skin concern. Each subplot corresponds to one concern, and bars show aggregated mutation counts for the genetic markers (MMP-1, MMP-3, SOD2, GPX, AQP3, and FLG) within each severity category.

For each candidate setting, clustering stability is estimated using repeated subsampling of the outer-training data. Specifically, we drew $R{=}10$ subsamples (STAB_REPEATS= 10), each containing 80% of the outer-training observations (SUBSAMPLE_FRAC= 0.80), fit k-modes on each subsample, and then *projected* each subsample solution to the *same reference set* by assigning labels to the full outer-training set using the learned modes. Stability is quantified as the mean pairwise Adjusted Rand Index (ARI) across the resulting $R$ labelings on the outer-training set, yielding an internal estimate of reproducibility under perturbations of the training data. In addition, we tracked a mismatch-based cost on the outer-training set, computed as the mean number of feature-wise mismatches between each sample and its assigned mode (lower is better).

Hyperparameters are selected by maximizing a $k$-penalized stability score to discourage over-fragmentation when stability improvements are marginal. Let $\overline {\mathrm {ARI}}$ denote the mean pairwise ARI across repeats for a candidate with $k$ clusters, and let $k_{\max }=5$ be the largest candidate value in the grid. The selection score is defined as

\begin{equation*} \mathrm {Score}(k) = \overline {\mathrm {ARI}} - \alpha \frac {k}{k_{\max }}, \tag {1}\end{equation*}

with $\alpha = 0.01$ (K_PENALTY_ALPHA= 0.01). When candidates produced similar scores, the mismatch cost is used as a secondary criterion, favoring lower-cost solutions.

In this study, the penalty term equals $0.002k$ since $\alpha /k_{\max }=0.01/5$.

After selecting hyperparameters, k-modes is refit on the full outer-training data to learn cluster prototypes (modes). Cluster labels for the held-out fold are then assigned using these training-derived prototypes only. This design yields fold-specific surrogate labels for downstream prediction while preserving strict separation between training and evaluation partitions. Performance and interpretability estimates are subsequently summarized as mean ± std across outer folds.

For visualization and descriptive reporting, we additionally refit k-modes on the full dataset using the modal hyperparameters that we select across the outer folds. We use this final refit only for visualization, whereas all reported performance and feature-attribution results come from the nested cross-validation protocol.

### E. Predictive Modeling and Feature Importance Analysis

This section evaluates **Hypothesis 3**, which posits that integrating genetic and lifestyle information provides superior predictive utility for dermatological profile assignment compared with either modality alone. Specifically, a supervised classifier is trained to predict cluster membership derived within each outer fold (Fig. 3). To align with a clinically actionable input scenario and to avoid trivial reconstruction of phenotype-driven clusters, the predictive models used genetics and lifestyle variables as inputs, while phenotype variables are excluded from the classifier feature set.

Model training and evaluation followed the outer 5-fold protocol. For each outer fold, the classifier is trained only on the outer-training split using the corresponding train-derived cluster labels and evaluated only on the held-out fold using labels assigned from the train prototypes. Performance is summarized using accuracy and macro-averaged precision, recall, and F1-score to account for potential class imbalance. We report results for a gradient-boosted tree classifier (XGBoost) under the same evaluation design.

To directly test **Hypothesis 3**, three feature configurations are evaluated under identical outer folds: (1) genetic markers only, (2) lifestyle/environmental factors only, and (3) the integrated genetics + lifestyle model. Improvements observed for the integrated configuration are interpreted as evidence that combining intrinsic (genetic) and extrinsic (lifestyle) factors yields complementary information for predicting dermatological profiles.

Model interpretability is assessed using fold-aware feature attribution and summarized as mean ± std across outer folds. We report (i) model-internal gain-based importance from XGBoost and (ii) permutation importance computed on the held-out fold within each outer iteration. In addition, SHAP-style feature contributions are computed from the trained XGBoost models on held-out data using XGBoost’s built-in contribution scores (pred_contribs), and stability of feature drivers is quantified by aggregating mean absolute contributions across folds. Collectively, these analyses identify consistent genetic and lifestyle determinants of profile assignment and support transparent interpretation suitable for clinical translation.

Overall, the proposed framework links unsupervised dermatological phenotyping to supervised prediction and explanation under a strictly partitioned nested cross-validation protocol. This enables robust estimation of predictive performance and stable identification of influential genetic and lifestyle factors supporting individualized dermatological profiling.

## III. Results

This section presents the findings of the study in a structured manner, encompassing descriptive genetic trends, statistical associations, gene–environment interactions, clustering patterns, and predictive modeling outputs.

### A. Descriptive Genetics Results

This section begins by characterizing the distribution and severity-related trends of genetic mutations across six major skin phenotypes. By analyzing aggregate mutation burden and stratifying by severity levels, we aim to uncover gene-specific patterns that may underlie key dermatological traits such as acne, dryness, and sensitivity. The goal is to explore whether increasing mutation load corresponds to worsening phenotype expression, and how this relationship varies across different genes. These descriptive analyses serve as a foundation for the subsequent inferential tests and predictive modeling, providing context for understanding how genetic variation contributes to inter-individual differences in skin health.

#### 1) Correlation Analysis

To investigate the genetic contribution to dermatological phenotypes, we first analyzed the aggregate and average mutation levels across severity grades for the six phenotypes: Acne, Dryness, Pigmentation, Redness, Scarring, and Sensitivity. We generate bar plots for each phenotype, stratified by severity level and gene (Fig. 4).

**FIGURE 5.**
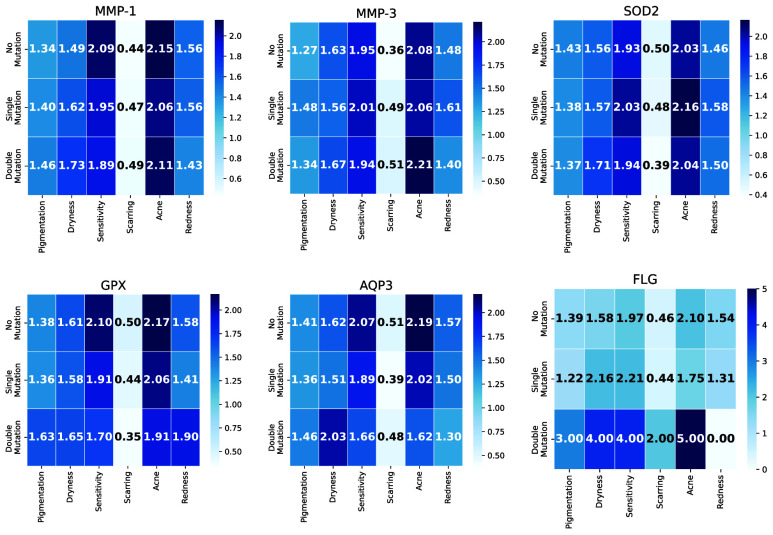
Heatmaps illustrating cumulative skin phenotype severity by gene-specific mutation status. Rows correspond to mutation status (none, single, double), and columns correspond to the six phenotypes, with color intensity indicating the summed severity scores.

The stratified analysis of mutation burden across skin concern severity levels revealed distinct genetic patterns:

• **Acne:** Mutation counts increase significantly with severity, particularly for *MMP-3* and *SOD2*, indicating a potential genetic contribution to inflammatory and oxidative pathways in acne pathogenesis.
• **Dryness:** Peak mutation levels are observed in the dry_moderate and dry_severe groups. Notably, *FLG* mutations, despite lower overall frequency, exhibited higher average levels in severe dryness, underscoring its established role in epidermal barrier function.
• **Pigmentation:** A progressive accumulation of mutations is evident from pigm_no to pigm_severe, with *MMP-3*, *GPX*, and *SOD2* emerging as key contributors, suggestive of cumulative genetic influence on melanogenesis or oxidative damage.
• **Redness:** Elevated mutation counts in red_no and red_mild categories suggest baseline genetic load. However, pronounced increases in *MMP-1*, *MMP-3*, and *SOD2* in higher severity groups point to their involvement in vascular and inflammatory responses.
• **Scarring:** Severity-associated increases in *MMP-1*, *MMP-3*, and *SOD2* mutation levels implicate these genes in extracellular matrix remodeling and impaired tissue repair mechanisms.
• **Sensitivity:** Substantial increases in *SOD2*, *MMP-1*, and *FLG* mutations in sensitive_moderate and sensitive_severe groups reinforce the role of oxidative stress and compromised barrier integrity in heightened skin reactivity.

These patterns collectively highlight the central roles of *MMP-1*, *MMP-3*, and *SOD2* across multiple skin phenotypes. The observed escalation of mutation burden with severity, particularly in acne, pigmentation, and sensitivity, underscores the genetic underpinnings of these traits and supports the integration of mutation profiling in personalized dermatological assessment.

#### 2) Mutation Frequency and Gene-Level Aggregation

To further investigate gene-specific contributions to skin concern severity, we implement a structured aggregation framework. We aggregate total phenotype severity for each gene at each mutation level (none, single, double) to create a heatmap visualization (Fig. ??), that illustrates how mutation profiles are associated with cumulative phenotypic burden.

These heatmaps (Figure 5) reveal distinct patterns in phenotype severity across genes and mutation categories. Sensitivity and acne consistently exhibited the highest cumulative severity scores, while scarring appeared as the least affected phenotype across all gene–mutation combinations. For most genes—*MMP-1*, *MMP-3*, *SOD2*, *GPX*, and *AQP3*—severity levels generally increased with mutation burden, with double mutations often associated with equal or higher phenotype scores compared to single mutations. This trend is consistent with a dose–response relationship, where increased mutation load may exacerbate phenotypic expression. However, isolated exceptions are observed where single mutations exhibited similar or even greater severity than double mutations, which could reflect sample size imbalances, non-additive genetic effects, or compensatory biological mechanisms. Notably, *FLG* displayed a distinct pattern, with double mutations clearly linked to markedly increased severity in acne and sensitivity. This finding aligns with its well-established role in maintaining epidermal barrier function, where loss-of-function mutations are known to impair skin integrity and increase susceptibility to inflammation and moisture loss.

**FIGURE 6.**
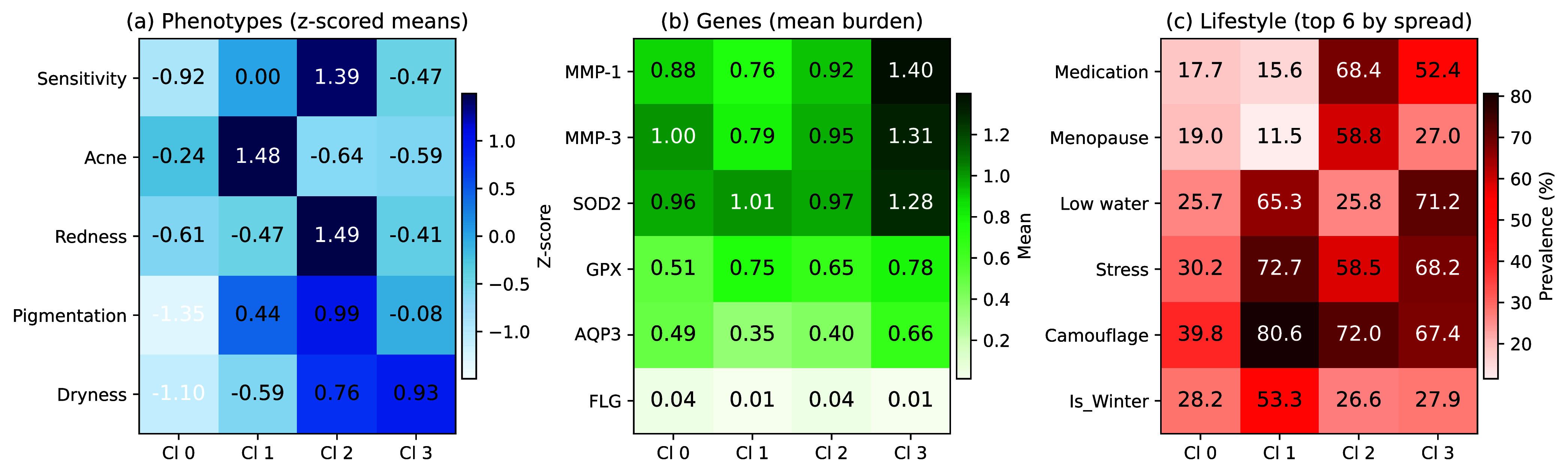
Cluster-wise profiles derived from the final $k{=}4$ k-modes solution used for descriptive profiling. Panel (a) shows phenotype means standardized across clusters (z-scored). Panel (b) shows mean genetic burden by gene. Panel (c) shows the top six lifestyle factors ranked by between-cluster spread, reported as prevalence (%) for binary variables and mean level for ordinal variables.

At the gene level, the following trends are observed:

• *MMP-1* and *MMP-3* are associated with elevated acne, dryness, and sensitivity scores, but showed limited impact on scarring.
• *SOD2* contributed to moderate yet consistent increases in severity across all phenotypes, reinforcing its role in oxidative stress regulation.
• *GPX* followed a similar trend to *SOD2*, albeit with slightly attenuated effects.
• *AQP3* showed strong associations with acne, dryness, and sensitivity, suggesting its role in hydration and cellular osmotic balance.
• *FLG* exhibited phenotype-specific effects, with double mutations corresponding to marked severity increases in acne and sensitivity.

Overall, the analysis underscores the nuanced impact of individual genetic mutations on dermatological traits. In most cases, single mutations appeared more consequential than double mutations—except for *FLG*, where a clear dose–response pattern is evident. Scarring emerged as the phenotype least influenced by genetic mutations, suggesting a greater contribution from environmental or post-inflammatory processes. By contrast, acne, dryness, and sensitivity exhibited the highest severity across multiple genes, reinforcing their polygenic basis. Furthermore, some gene–phenotype relationships are more specific: for instance, *AQP3* is particularly associated with acne severity, whereas *FLG* is more strongly linked to both sensitivity and redness.

These findings highlight the complex interplay between gene-specific effects and mutation dosage, offering deeper insight into how inherited variation contributes to diverse dermatological outcomes.

### B. Statistical Results

#### 1) Genetic Mutation Burden and Skin Phenotype Severity

We employ one-way ANOVA to examine whether skin phenotype severity significantly differs among individuals grouped by their genetic mutation burden—categorized as no mutation, single mutation, or double mutation. The ANOVA results reveal statistically significant differences across all six phenotypes ( ${p}~< 0.05$; see Table 2). The strongest associations are observed for sensitivity ( ${p}=0.0255$), acne ( ${p}=0.0307$), and dryness ( ${p}=0.0402$), indicating that mutation count exerts a meaningful influence on phenotype expression.

**TABLE 2:** ANOVA $p$-Values for Severity Differences Across Mutation Levels

Skin Concern	ANOVA $p$-value
Pigmentation	0.0377
Dryness	0.0402
Sensitivity	0.0255
Scarring	0.0372
Acne	0.0307
Redness	0.0307

To identify which groups primarily contribute to these differences, we conduct post-hoc pairwise $t$-tests. The most pronounced differences are observed between individuals with double mutations and those with either no or single mutations. Notably, pigmentation ( ${p}=0.023$), sensitivity ( ${p}=0.016$), and acne ( ${p}=0.019$) demonstrate consistent and significant severity increases among double mutation carriers (Table 3). Comparisons between the no-mutation and single-mutation groups are largely non-significant, supporting the presence of a threshold effect in which phenotypic severity increases sharply in individuals carrying two risk alleles.

**TABLE 3:** Pairwise $t$-Test $p$-Values for Severity Differences Across Mutation Levels

Skin Concern	No vs. Single	No vs. Double	Single vs. Double
Pigmentation	0.561	0.0234	0.0201
Dryness	0.518	0.0254	0.0239
Sensitivity	0.477	0.0164	0.0171
Scarring	0.512	0.0223	0.0233
Acne	0.483	0.0193	0.0205
Redness	0.528	0.0192	0.0176

These results underscore a dose-dependent relationship between genetic mutation burden and phenotype severity. In particular, double mutation carriers consistently reported higher severity for conditions such as pigmentation, acne, and sensitivity. This threshold effect—observed only in individuals with homozygous or compound heterozygous mutations—suggests that cumulative genetic load may act as a tipping point in the manifestation of dermatological traits. These findings confirm **Hypothesis 1**, which posited that individuals with a greater burden of mutations in skin-related genes exhibit more severe dermatological phenotypes. The consistent pattern of increased severity among double mutation carriers across multiple skin concerns supports a dose-dependent genetic contribution to skin health.

The strongest associations are evident in phenotypes linked to skin barrier integrity and inflammation. For instance, mutations in the *FLG* gene—critical for epidermal differentiation and moisture retention—are closely aligned with increased sensitivity and dryness, corroborating prior findings implicating *FLG* in barrier dysfunction and atopic dermatitis [40].

Similarly, elevated acne severity in carriers of antioxidant-related gene mutations, such as *SOD2* and *GPX*, supports the hypothesis that oxidative stress plays a central role in acne pathogenesis. This is reinforced by clinical studies reporting increased lipid peroxidation and reduced antioxidant enzyme activity among acne patients [41]. These findings suggest that reduced antioxidant capacity may exacerbate inflammatory responses under environmental or hormonal stressors.

Variation in pigmentation and redness severity associated with *MMP-1* and *MMP-3* polymorphisms reflects the broader influence of extracellular matrix remodeling on visible skin traits. These metalloproteinases regulate collagen degradation and dermal architecture—processes known to be modulated by both genetic predisposition and environmental exposure such as ultraviolet radiation [42].

Collectively, these statistical outcomes provide strong empirical support for the inclusion of genetic mutation profiles in predictive dermatological modeling. They also affirm the biological plausibility of incorporating such features in AI-based personalized skincare frameworks. By accounting for genotypic variation alongside phenotype heterogeneity, these findings offer robust validation for Hypothesis 1, confirming that mutation burden significantly contributes to inter-individual differences in skin concern severity.

#### 2) Gene–Environment Interactions on Skin Phenotypes

To statistically validate the observed gene–environment interactions, we perform a series of two-way ANOVA tests for five biologically motivated gene $\times $ lifestyle combinations, as detailed in the Methods section. This modeling approach enables us to quantify whether the effect of a lifestyle factor on skin phenotype severity significantly differs between genetic mutation carriers and non-carriers.

Among the tested interactions, three showed statistically significant effects. *AQP3* mutation carriers reported significantly higher dryness scores in winter compared to non-carriers (interaction $F(1, 5250) = 32.43$, $p < 0.000001$), confirming the hypothesized synergy between impaired hydration mechanisms and cold weather exposure. Similarly, pigmentation severity is significantly greater among medicated individuals with *GPX* mutations ( $F(1, 5250) = 8.16$, $p = 0.0043$), supporting the idea that oxidative stress or photosensitization from treatment compounds may be amplified in genetically predisposed individuals. Additionally, redness scores are significantly higher among individuals with *MMP-3* mutations who also resided in urban environments ( $F(1, 5250) = 8.86$, $p = 0.003$), suggesting a potential exacerbating role of pollution and environmental stress on extracellular matrix degradation in genetically susceptible individuals.

Other tested hypotheses, including *FLG* $\times $ Scrub Usage $\rightarrow $ Sensitivity and *SOD2* $\times $ Stress $\rightarrow $ Acne, could not be conclusively evaluated due to sparse subgroups or non-significant interaction terms. Nonetheless, these results reinforce the presence of gene–environment interactions and complement the clustering patterns observed in unsupervised analysis.

These results affirm that genotype–phenotype relationships can be meaningfully modulated by lifestyle factors, supporting the development of personalized, context-aware dermatological interventions. Although multiple statistical tests are performed across six phenotypes and five gene–environment hypotheses, we do not apply formal multiple-comparison corrections such as Bonferroni or false discovery rate adjustments. Given the hypothesis-driven design and the strong biological rationale supporting each test, we treat ANOVA and interaction models independently and interpret significance at the conventional $p~< ~0.05$ threshold. Importantly, most of the significant findings—including those involving *FLG*, *MMP-3*, *GPX*, and *AQP3*—yield p-values well below 0.01, mitigating concerns of Type I error. This approach is consistent with recent statistical guidance that emphasizes thoughtful interpretation of p-values in context rather than rigid adherence to correction thresholds [43]. Nonetheless, results near the threshold are interpreted cautiously and may warrant replication in future studies.

### C. Clustering Results

k-modes clustering is used to derive discrete dermatological profiles from integrated categorical features (genetics, lifestyle, and phenotypes). To prevent circularity when using cluster membership as a downstream supervised target, all clustering selection and evaluation follow a leakage-free nested cross-validation design (Fig. 3). Specifically, k-modes hyperparameters are selected exclusively on the outer-training split using a stability criterion (Adjusted Rand Index, ARI, across repeated subsamples) and mismatch cost; cluster labels for the held-out outer fold are then assigned using only the training-derived prototypes. The inner-loop selections are summarized in Table 4, showing consistent stability (mean ARI $\approx 0.74$) and modest variation in the selected $k$ across folds.

**TABLE 4:** Nested CV Inner-Loop k-Modes Selection on Outer-Train Only

Fold	$k$	init	$n_{\text {init}}$	max_iter	ARI	Cost	Score
1	5	Cao	5	100	0.805	9.584	0.695
2	4	Cao	5	100	0.703	9.846	0.595
3	4	Cao	5	100	0.729	9.790	0.621
4	3	Cao	5	100	0.746	9.989	0.640
5	4	Cao	5	100	0.723	9.747	0.615
Mean ± Std	3–5	Cao	5	100	$0.741~\pm ~0.035$	$9.791~\pm ~0.132$	$0.633~\pm ~0.034$

For descriptive profiling and visualization, a final k-modes model is refit on the full dataset using $k{=}4$ (the modal choice across folds) to produce a single, stable set of profiles for reporting cluster prevalence and interpretability figures. The resulting cluster prevalence is reported in Table 5. This refit is used only for descriptive plots and subgroup characterization; predictive performance is reported strictly from the outer folds (Section III-D).

**TABLE 5:** Final Cluster Prevalence for Visualization Using a $k=4$ k-Modes Refit

Cluster	Count	Percent
0	3070	58.4%
1	990	18.8%
2	728	13.9%
3	466	8.9%

Figure 6 summarizes cluster-wise patterns across the three input modalities. For interpretability, this figure is based on a *full-cohort descriptive* k-modes fit (k= 4) used only for profiling/visualization and *should not be interpreted as an evaluation result*; all model selection and generalization performance are reported from the leakage-free nested cross-validation pipeline (Fig. 3). A plain-English summary of lifestyle/environmental characteristics by cluster is provided in *Supplementary 7*. Phenotype means (standardized across clusters) indicate that Cluster 1 is acne-dominant, whereas Cluster 2 exhibits higher sensitivity/redness and concomitant dryness/pigmentation relative to the other profiles. Cluster 0 shows globally lower phenotype severity, consistent with a lower-risk reference profile. In parallel, genetic burden is most elevated in Cluster 3 (notably across MMP-1/MMP-3 and oxidative-stress related genes), while lifestyle factors differentiate clusters through distinct exposure/behavior signatures (e.g., hydration, stress, medication, and menopause prevalence). Together, these comparisons support that the inferred clusters correspond to interpretable dermatological *profiles* within this cohort, rather than minor variations of a single dominant pattern.

**FIGURE 7.**
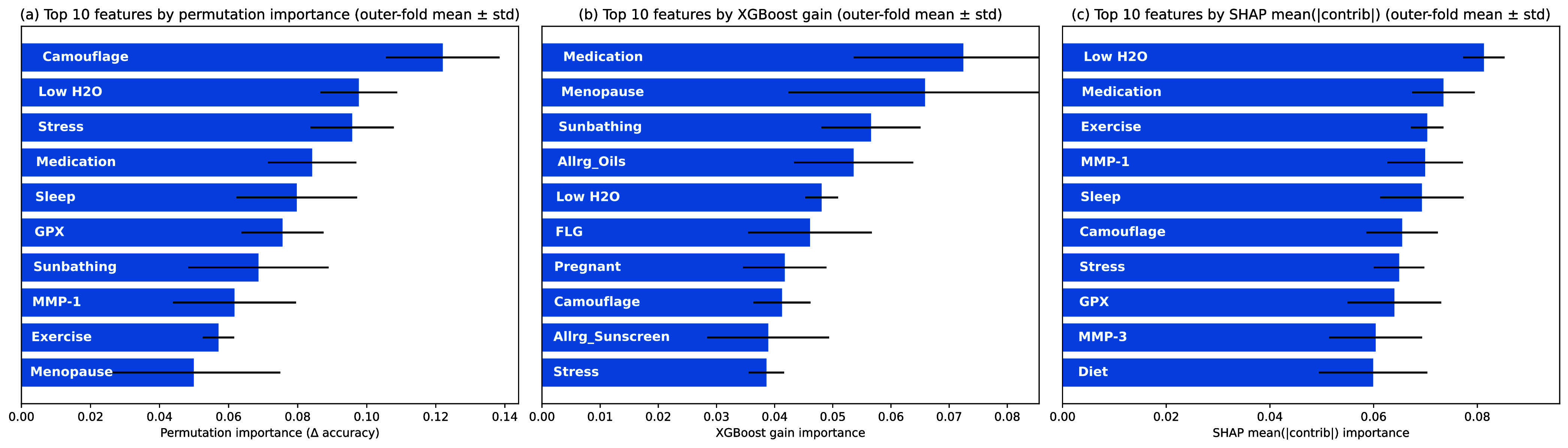
Top 10 features by (a) permutation importance computed on the outer-test fold, (b) XGBoost gain (model-internal), and (c) SHAP mean(—contrib—) summarized over outer-test predictions; values are reported as outer-fold mean ± std.

### D. Predictive Modeling and Feature Importance

Supervised learning is used to quantify how well genetics plus lifestyle recover the derived profile membership under leakage-controlled evaluation. Table 6 reports outer-fold performance for predicting cluster membership using genetics and lifestyle inputs, where test-fold labels are assigned using k-modes prototypes fit only on the corresponding outer-training split (Fig. 3). Performance remains high and consistent across folds, with mean accuracy $0.9789 \pm 0.0083$ and macro-F $1~0.9711 \pm 0.0126$, indicating robust prediction across both prevalent and minority clusters.

**TABLE 6:** Leakage-Free Nested CV Outer-Fold Performance for Cluster Prediction Using Genetics Plus Lifestyle Inputs

Outer Fold	Accuracy	Macro-F1	Macro-Prec.	Macro-Rec.
1	0.9762	0.9666	0.9709	0.9636
2	0.9677	0.9535	0.9439	0.9643
3	0.9791	0.9730	0.9821	0.9643
4	0.9905	0.9880	0.9913	0.9850
5	0.9810	0.9746	0.9789	0.9711
Mean ± Std	$0.9789~\pm ~0.0083$	$0.9711~\pm ~0.0126$	$0.9734~\pm ~0.0181$	$0.9697~\pm ~0.0091$

To test Hypothesis 3, we conduct a modality ablation under the same leakage-free outer-fold evaluation used throughout this study. Within each outer fold, we train an XGBoost classifier using three feature sets: (1) genetics only, (2) lifestyle/environment only, and (3) the integrated (genetics+lifestyle) set, while preserving fold-specific surrogate labels derived from training prototypes only. Table 7 reports mean±std performance across outer folds (macro-averaged metrics). The integrated model consistently outperforms either single-modality variant, supporting Hypothesis 3 that genetic and behavioral inputs provide complementary predictive value for cluster membership.

**TABLE 7:** Modality Ablation for Hypothesis 3 Under the Same Leakage-Free Outer-Fold Evaluation. Values are Mean ± Std Across Outer Folds (Macro-Averaged Metrics). The Integrated (Genetics+Lifestyle) Row Matches Table 6

Feature Set	Accuracy	Macro-F1	Macro-Prec.	Macro-Rec.
Genetics only	$0.8230~\pm ~0.0029$	$0.7970~\pm ~0.0109$	$0.8010~\pm ~0.0212$	$0.7940~\pm ~0.0362$
Lifestyle only	$0.8810~\pm ~0.0098$	$0.8610~\pm ~0.0101$	$0.8690~\pm ~0.0176$	$0.8530~\pm ~0.0677$
Integrated (Genetics+Lifestyle)	$0.9789~\pm ~0.0083$	$0.9711~\pm ~0.0126$	$0.9734~\pm ~0.0181$	$0.9697~\pm ~0.0091$

From a translational standpoint, the macro-averaged recall ( $0.9697 \pm 0.0091$) indicates that the model maintains sensitivity across all profiles, supporting identification of minority or higher-risk clusters rather than optimizing only the dominant group. In addition, the convergence of leading predictors across outer folds and across complementary attribution methods (permutation, gain, and SHAP; Fig. 7) improves interpretability and clinician trust, since the same small set of drivers repeatedly explains profile assignment under resampling. Finally, the integrated genetics+lifestyle input setting directly supports Hypothesis 3 and yields actionable counseling targets, where stable modifiable factors can be mapped to consistent intervention categories (e.g., exposure mitigation, barrier support, and routine adjustment).

**FIGURE 8.**
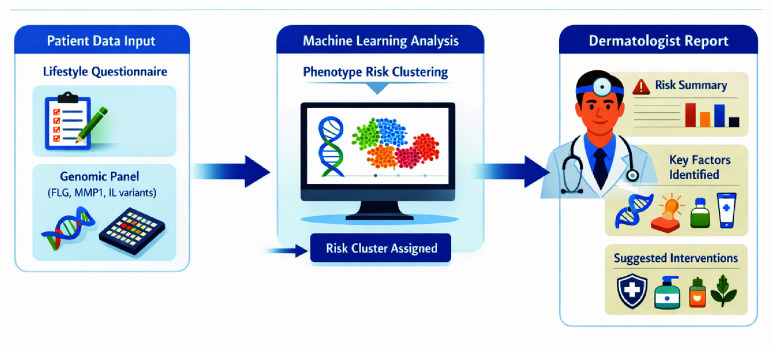
**Workflow integration for clinical decision support**. A patient lifestyle questionnaire and a targeted genomic panel are processed to assign a phenotype risk cluster. The dermatologist receives a concise report including a risk summary, key contributing factors, and intervention categories to support stratified counseling and prevention planning.

We additionally evaluated a leakage-free (non-nested) 5-fold design with fold-wise clustering, label assignment, and prediction. This produces comparable performance (accuracy $0.968 \pm 0.007$, macro-F $1~0.967 \pm 0.008$) and consistent leading predictors; the full results are provided in *Supplementary 8*.

To assess interpretability and stability of predictors under fold-wise evaluation, feature attribution is aggregated across outer folds using three complementary measures: (i) *XGBoost gain* (a model-internal split-importance computed from the trained model in each outer fold), (ii) *permutation importance* computed on the *outer-test* fold as the decrease in performance after feature shuffling, and (iii) *SHAP* mean absolute contribution computed from the trained XGBoost models and summarized over the *outer-test* fold predictions. Figure 7 reports the top 10 features under each attribution method (outer-fold mean ± std).

Figure 7 reports the top 10 features under each attribution method (outer-fold mean ± std). Across methods, several modifiable lifestyle/exposure variables recur as stable drivers (e.g., camouflage usage, hydration/low-water behavior, stress, sleep, medication, and menopause), while genetic markers related to oxidative stress and matrix remodeling (e.g., *GPX*, *MMP-1*, and *MMP-3*) also appear among the most influential predictors. The agreement in the leading signals across attribution methods supports their use as stable candidates for downstream clinical interpretation and decision support.

## IV. Discussion

This study demonstrates the multifactorial nature of skin health by integrating genetic mutation profiles, lifestyle exposures, and self-reported skin phenotypes from a large real-world dataset. Our findings show that skin conditions do not arise from single causes but rather emerge from interactions between intrinsic genetic vulnerabilities and modifiable behavioral and environmental factors. The combination of unsupervised clustering and supervised modeling enables the identification of four distinct dermatological subtypes with high predictive accuracy under leakage-free nested cross-validation, underscoring the potential of interpretable machine learning for personalized skincare applications.

Our results validate the three study hypotheses. First, consistent with Hypothesis 1, higher mutation burden in skin-relevant genes such as *FLG*, *MMP-3*, and *SOD2* is associated with increased severity of dryness, sensitivity, and other phenotypes. This finding reinforces prior research linking impaired barrier function, oxidative stress dysregulation, and matrix remodeling to these gene variants [44], [45], [46]. Second, Hypothesis 2 is supported by statistically significant gene–environment interactions, including *GPX* $\times $ Medication Use (Pigmentation) and *AQP3* $\times $ Winter Exposure (Dryness), as well as *MMP-3* $\times $ City Living (Redness). These effects indicate that lifestyle and environmental exposures can amplify or modulate genetic risk, motivating context-aware personalization rather than genotype-only stratification. Third, Hypothesis 3 is supported by the integrated genetics+lifestyle model achieving strong and stable outer-fold performance (accuracy $\approx 0.98$; macro-F $1~\approx 0.97$), supporting the value of multimodal modeling for dermatology-oriented risk stratification.

Rather than reiterating each cluster’s characteristics, we highlight clinically interpretable patterns supported by the cluster-wise profiling results (Fig. 6; see also *Supplementary 7*). Cluster 0 represents a lower-severity reference profile with comparatively favorable phenotype patterns. Cluster 1 is acne-dominant, distinguishing a subgroup where modifiable exposures and routines are likely to be salient for counseling and prevention planning. Cluster 2 exhibits a reactive/inflammatory profile with elevated sensitivity and redness (and higher pigmentation relative to other clusters), while Cluster 3 is dryness-dominant, aligning with barrier-supportive management priorities. Importantly, these clusters are not defined by phenotypes alone: cluster-wise summaries show structured differences across both genetic burden and lifestyle exposures, supporting that the inferred clusters correspond to interpretable dermatological profiles rather than minor variations of a single dominant pattern.

Our findings also align with and extend previous literature. The link between *FLG* mutations and dry, sensitive skin is widely documented [47], and we show this signal remains detectable in a real-world cohort while simultaneously modeling lifestyle covariates. Similarly, matrix metalloproteinase burden (e.g., *MMP-3*) aligns with remodeling-related outcomes such as scarring, echoing prior work on metalloproteinase involvement in wound repair and dermal turnover [42]. Lifestyle factors relevant to oxidative stress and exposure management also emerge as important predictors, consistent with established roles of behavioral and environmental influences in skin physiology [41], [48]. By integrating these variables in a leakage-controlled machine learning framework, our study complements traditional dermatological research and provides a pathway toward actionable, AI-powered phenotyping.

Feature importance analyses strengthen interpretability by demonstrating convergence of leading predictors across complementary attribution methods (permutation importance, XGBoost gain, and SHAP mean absolute contribution). Across outer folds, several modifiable lifestyle/exposure variables recur among the most influential drivers of profile membership, including hydration-related behavior (Low^.^H2O), medication status, stress, sleep, camouflage usage, menopause status, exercise, and sunbathing, while genetic markers (notably *GPX*, *MMP-1*, *MMP-3*, and *FLG*) also appear among the stable predictors. This agreement across methods supports clinician trust because it indicates that key drivers are not artifacts of a single importance technique. In practical terms, fold-stable factors enable consistent counseling targets (e.g., hydration behavior, stress/sleep hygiene, exposure mitigation) while preserving a genetic susceptibility context for individualized prevention planning. We note that while exfoliation-related factors are biologically plausible modifiers for barrier-compromised individuals, the FLG $\times $ scrub interaction is not conclusively supported in the present two-way ANOVA results and warrants further investigation in prospective or enriched cohorts.

Overall, the proposed framework demonstrates that leakage-controlled clustering-derived profiles can be predicted with high macro-averaged performance using genetics and lifestyle inputs, while producing consistent attribution summaries across folds. The profiles and drivers should be interpreted as hypothesis-generating and require external validation on clinician-adjudicated endpoints and more diverse cohorts before clinical deployment. Nonetheless, the workflow naturally supports decision-support style reporting (profile membership, confidence, and top contributing factors) that can be integrated into counseling and follow-up pathways.

### A. Clinical Endpoints and Decision Support Implications

A key translational objective of this work is to move beyond technical performance metrics and specify how integrative dermatogenomic modeling can support clinically meaningful endpoints. The proposed framework assigns each individual to an interpretable phenotype risk cluster derived from multi-domain signals (genetics, lifestyle, and engineered phenotype grades). This cluster assignment can be operationalized as a decision-support output that supports cohort-calibrated risk profiling and targeted counseling rather than replacing clinical diagnosis. In routine dermatology care, the primary clinical endpoints supported by this framework include: (i) *risk stratification* for barrier dysfunction and reactive skin phenotypes (e.g., elevated sensitivity, dryness, and redness), (ii) *early identification* of individuals trending toward high-risk phenotypes before symptom escalation, and (iii) *personalized prevention planning* by linking modifiable lifestyle exposures to cluster-specific risk signatures. These endpoints are aligned with practical care pathways in which prevention, trigger avoidance, and personalized skincare routines aim to reduce flare frequency and improve patient-reported outcomes.

Figure 8 illustrates the intended system-level integration of the proposed approach within a clinical decision-support workflow. At the point of intake, the patient provides a structured lifestyle questionnaire alongside a targeted genomic panel (e.g., *FLG*, *MMP1*, and other skin-relevant variants; expandable as additional markers become available). The model processes these inputs to assign a phenotype risk cluster and generates an accompanying explanation layer. The dermatologist (or trained clinician) receives a concise report that includes (i) a risk summary, (ii) the dominant contributing factors driving cluster assignment, and (iii) suggested intervention categories. In practice, these suggested categories map to common clinical actions such as barrier-supportive regimens for dryness-prone/reactive profiles, counseling on hydration and routine adjustment, and exposure mitigation strategies. This structure supports efficient triage and consistent documentation while preserving clinician oversight and individualized judgment.

**Comparative value over single-factor assessment.** The decision-support value of the integrative approach is that it combines intrinsic susceptibility and modifiable exposures within a unified predictive and explainable framework. Genetics-only risk scores are informative for predisposition but typically provide limited guidance on near-term management because they do not capture behavioral triggers or exposure patterns that shape phenotype expression. Conversely, lifestyle-only questionnaires can identify triggers but do not account for genetically mediated vulnerability or differential responsiveness to interventions. By integrating genetic and lifestyle factors, the model supports *Hypothesis 3* at the translational level: multi-domain inputs enable more accurate and actionable stratification than either modality alone. Importantly, the explanatory layer (fold-stable drivers across permutation, gain, and SHAP) provides transparency for clinical users, allowing the reported risk cluster and its primary determinants to be reviewed and discussed with the patient during counseling.

**Clinical decision support scope.** The output of the system is interpreted as a structured risk profile that augments clinical reasoning. It standardizes intake, prioritizes high-risk individuals for earlier follow-up, and tailors prevention strategies around the most salient drivers identified for each patient. Future prospective evaluation should assess the impact on endpoints such as reduction in flare frequency, improved symptom severity scores, and patient-reported quality-of-life measures, as well as workflow metrics such as documentation completeness and time-to-decision during consulta-

tion.

### B. Limitations and Future Work

This study presents several limitations that warrant consideration. First, the dataset is derived from a proprietary cohort comprising predominantly middle-aged female participants, which may limit the generalizability of findings to other demographic groups. Future research should seek to validate these results across more diverse populations, including varying age ranges, genders, and ethnic backgrounds.

Second, skin phenotype severity is based on self-reported survey responses, which may be subject to reporting bias and variability in interpretation. Incorporating clinician-assessed or image-derived phenotype data may improve the robustness and objectivity of the model outputs.

Third, the genomic analysis is limited to six preselected skin-related genes. While this candidate-gene approach facilitates biological interpretability, it does not account for potential polygenic or novel associations. Future studies should consider the integration of genome-wide data to capture additional predictive markers and interactions.

Additionally, the cross-sectional design of the dataset precludes longitudinal analysis. Prospective studies could assess changes in skin phenotypes over time, particularly in response to lifestyle modifications or targeted interventions.

Finally, while the present framework already includes fold-aware permutation and SHAP-based explanations, future work should further strengthen transparency at the individual level (e.g., patient-specific explanation summaries and counterfactual “what-if” recommendations) and evaluate real-world deployment considerations, including calibration, fairness across subgroups, and integration with digital intake systems and clinical documentation.

## V. Conclusion

In this study, we developed and evaluated an integrative AI framework that combines genetic, phenotypic, and lifestyle data to derive and predict dermatological risk profiles. The pipeline is intended to support personalized skincare recommendations and decision support using routinely obtainable intake data. Our clustering analysis identified four interpretable skin health subtypes, each characterized by distinct combinations of genetic burden and behavioral factors. Using a leakage-free nested cross-validation protocol, we achieve high and consistent prediction of profile membership from genetics plus lifestyle inputs using an XGBoost classifier (mean accuracy $\approx 0.979$ across outer folds), supporting robust generalization under strict train–test separation.

Our findings underscore the importance of multi-modal modeling in dermatogenomics. Statistically significant associations between mutation burden and phenotype severity, together with meaningful gene–environment interactions, highlight the complex interplay underlying individual skin health. Moreover, fold-aggregated explainability analyses (permutation importance and SHAP contributions) identify stable and actionable drivers, supporting individualized counseling targets such as exposure mitigation, routine adjustment, and lifestyle-related risk modification.

We acknowledge limitations, including demographic imbalance in the cohort, reliance on self-reported phenotype severity, and the restricted scope of the six-gene panel. Nonetheless, the results provide a proof-of-concept for integrating genetic susceptibility with modifiable exposures in an explainable ML workflow for precision dermatology.

Looking ahead, we envision expanding this framework to incorporate additional molecular markers, longitudinal follow-up, and objective clinical or imaging-based endpoints to strengthen validity and clinical utility across diverse populations. Improving interpretability, calibration, and deployment integration (e.g., structured intake and report generation) will further advance scalable and personalized dermatological risk stratification driven by combined genetic and lifestyle insight.

## Data Availability Statement

The data used in this study are proprietary and are provided by Nomige.com, a company specializing in personalized skincare solutions based on genetic and lifestyle data. Raw genotype or phenotypic data cannot be used due to limitations imposed by ethics.

## Conflict of Interest

This study used data provided by Nomige, a company specializing in personalized skincare. Barbara Geusens is the founder of Nomige. The other authors declare that they have no competing interests.

## Supplementary Materials

Supplementary Materials
