# Supplementary material for: Integrative Machine Learning of Genetic and Lifestyle Factors for Personalized Skin Health
Source: IEEE J Transl Eng Health Med. 2026 Mar 19;14:164–78. doi: 10.1109/JTEHM.2026.3675676 (PMC13068124; doi:10.1109/JTEHM.2026.3675676)
Supplement: Supplementary Materials [file supp1-3675676.pdf]

SUPPLEMENTARY 1: DATASET ATTRIBUTES

Supplementary Table 1: Overview of Dataset Columns

Category	Columns
Genetics	MMP-1, MMP-3, SOD2, GPX, AQP3, FLG
Phenotype	Pigmentation, Dryness, Sensitivity, Scarring, Acne, Redness
Lifestyle	(Divided into subcategories below)

Supplementary Table 2: Lifestyle Attributes

Category	Variable	Description / Possible Values
**Health & Habits**	Pregnant	Indicates if the person is pregnant: 0 = Yes, 1 = No
	Menopause	Indicates menopause status: 0 = No, 1 = Yes
	Dietary Habits	Frequency of healthy/restricted diet: 0 = Rarely, 1 = Often, 2 = Most of the time, 3 = Frequently
	Exercise	Weekly physical activity: 0 = Rarely ($< 1h$), 1 = Sometimes (1h), 2 = Often (2–3h), 3 = Almost always ($> 3h$)
	Sleeping Habits	Restful sleep frequency: 0 = Rarely, 1 = Often, 2 = Most of the time, 3 = Frequently
	Medication	Takes medications: 0 = No, 1 = Yes
	Allergies	Contact allergies – Oils, Perfume, Nickel, Wool, Sunscreen: each coded as 0 = No, 1 = Yes
**Environmental Factors**	Sunbathing	Frequency of sun exposure: 0 = Rarely, 1 = Often, 2 = Most of the time, 3 = Frequently
	Living Outdoors	Outdoor time/day: 0 = $< 1h$, 1 = 1–2h, 2 = 2–4h, 3 = $> 4h$
	City_Living	Lives or works in big city: 0 = No, 1 = Yes
	Is_Winter	Current season is winter: 0 = No, 1 = Yes
	Fly_AC_YN	Frequent flyer / daily A/C exposure: 0 = No, 1 = Yes
**Skincare & Hygiene**	Scrub_Usage	Uses granular scrub $> 1/week$: 0 = No, 1 = Yes
	Camouflage Imperfections	Uses makeup to hide skin flaws: 0 = No, 1 = Yes
**Psychological & Behavioral**	Stress_Level	Stress state: 0 = Calm, 1 = Stressed
	Optimism_Level	Self-perception: 0 = Optimistic, 1 = Pessimistic
**Hydration & Smoking**	Low_H2O	Drinks $< 1.5L/day$: 0 = No, 1 = Yes
	Smoker	Smokes or smoked daily $\geq$1 year: 0 = No, 1 = Yes

SUPPLEMENTARY 2: SUMMARY OF KEY SKIN-RELATED GENES, THEIR BIOLOGICAL FUNCTIONS, AND SAMPLE COUNTS

Gene	Primary Function in Skin Health	Count
FLG	**Filaggrin**; essential for skin barrier formation and hydration; mutations are strongly associated with dryness and atopic dermatitis.	168
AQP3	**Aquaporin-3**; facilitates water and glycerol transport across keratinocyte membranes, supporting epidermal hydration and barrier recovery.	2440
GPX1	**Glutathione peroxidase 1**; antioxidant enzyme that reduces hydrogen peroxide and lipid hydroperoxides, protecting skin from oxidative stress.	3128
SOD2	**Superoxide dismutase 2**; mitochondrial antioxidant enzyme that neutralizes superoxide radicals, mitigating oxidative damage and inflammation.	5258
MMP1	**Matrix metalloproteinase-1**; degrades extracellular matrix components such as collagen, contributing to tissue remodeling and photoaging.	4784
MMP3	**Matrix metalloproteinase-3**; regulates extracellular matrix turnover and inflammatory responses, often linked to scarring and redness.	5158

Supplementary 3: Distribution of Categorical Responses for Skin Phenotype Questions

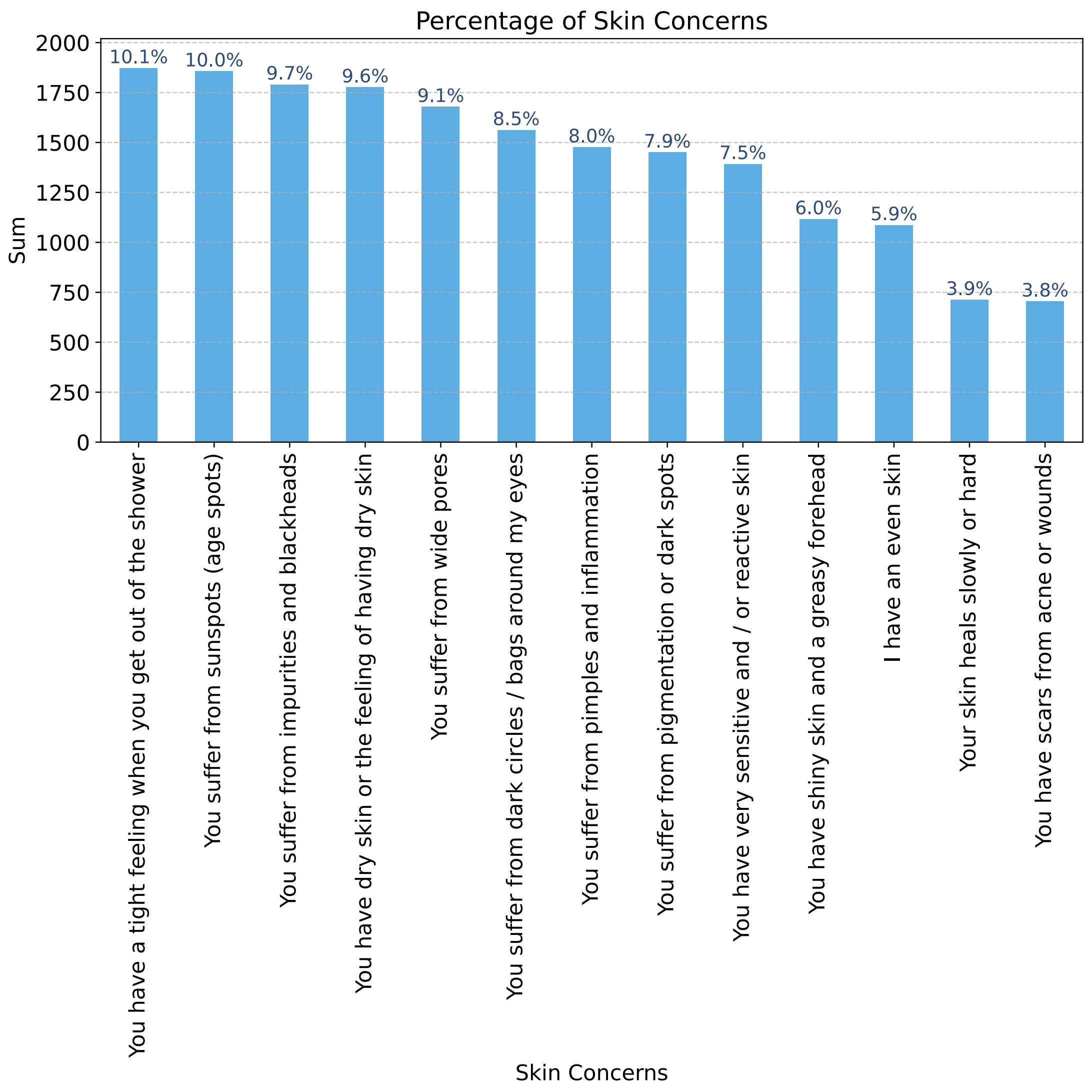

SUPPLEMENTARY 4: SKIN CONCERN ENCODING RULES

Concern	Severity Level	Description
**Acne**	mild	You suffer from wide pores
	mild to moderate	You have shiny skin and a greasy forehead
	moderate	You suffer from impurities and blackheads
	moderate to severe	I have subcutaneous bumps
	severe	You suffer from pimples and inflammation
**Pigmentation**	mild	You suffer from dark circles / bags around your eyes
	moderate	You suffer from sunspots (age spots)
	severe	You suffer from pigmentation or dark spots
**Dryness**	mild	Tight feeling after shower
	mild to moderate	Only cheeks are dry
	moderate	You have dry skin or the feeling of it
	moderate to severe	Skin is rough and flaky
	severe	Rash and skin often itches
**Sensitivity**	Not sensitive	Not indicated / Skin not sensitive
	mild	Reactive but not necessarily sensitive
	mild to moderate	Sometimes reacts to products
	moderate	Very sensitive and/or reactive skin
	severe	Reacts to almost all products tried
**Scarring**	mild	Skin heals slowly or with difficulty
	moderate	Scars from acne or wounds
	severe	Pits in the face

SUPPLEMENTARY 5: FULL ENCODING SCHEMA *(Severity Levels for Each Skin Phenotype)*

**Phenotype**	**Severity Levels**
Pigmentation	pigm_no — pigm_mild — pigm_moderate — pigm_severe
Dryness	dry_no — dry_mild — dry_mild_moderate — dry_moderate — dry_moderate_severe — dry_severe
Sensitivity	sensitive_no — sensitive_mild — sensitive_mild_moderate — sensitive_moderate — sensitive_severe
Scarring	scarring_no — scarring_mild — scarring_moderate — scarring_severe
Acne	acne_no — acne_mild — acne_mild_moderate — acne_moderate — acne_moderate_severe — acne_severe
Redness	red_no — red_mild — red_mild_moderate — red_moderate — red_moderate_severe — red_severe

SUPPLEMENTARY 6: DISTRIBUTION OF SEVERITY LEVELS ACROSS ALL SIX SKIN CONCERNS AFTER ENCODING

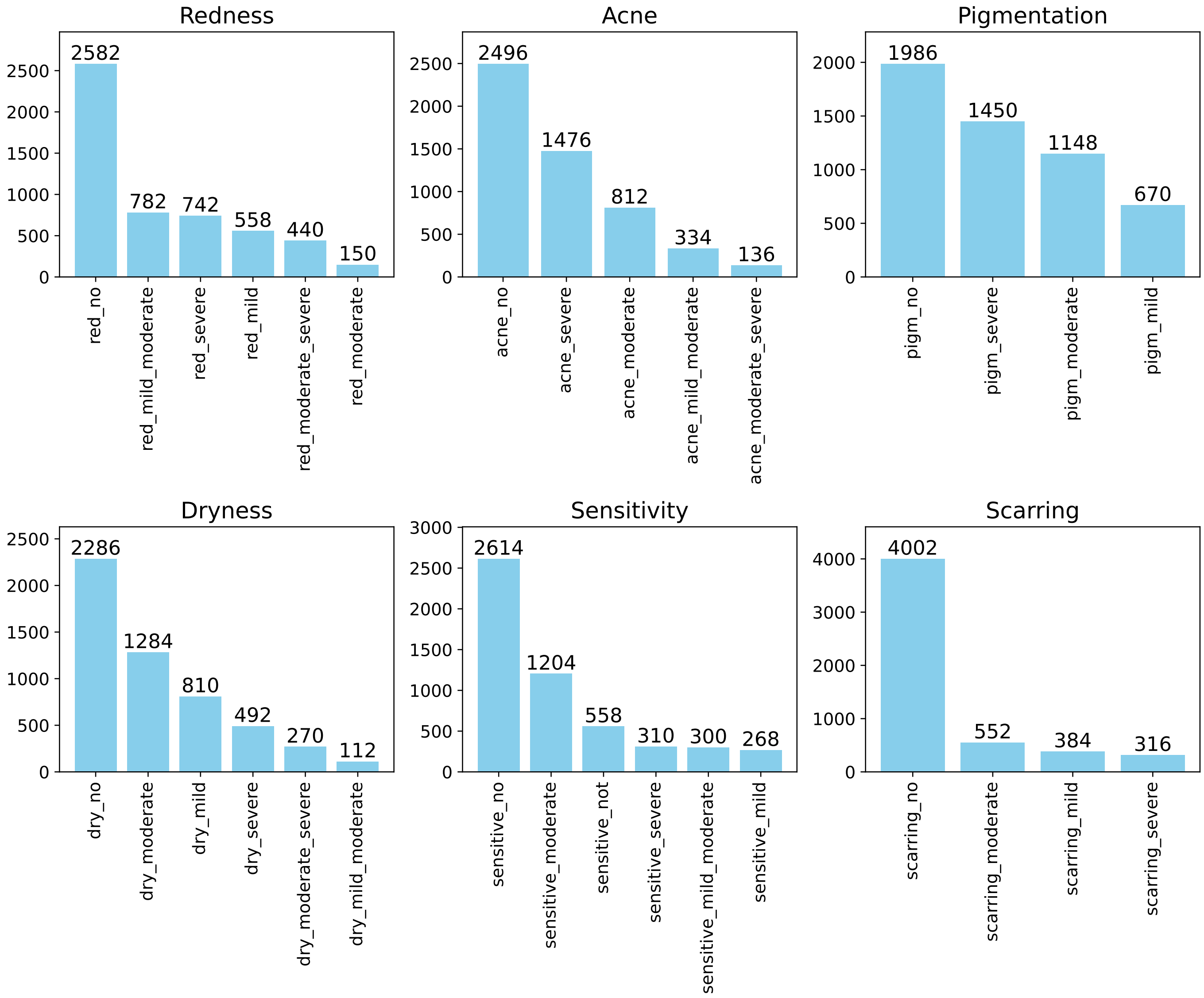

SUPPLEMENTARY 7: LIFESTYLE AND ENVIRONMENTAL CHARACTERISTICS ASSOCIATED WITH EACH DERMATOLOGICAL CLUSTER PROFILE

**Feature**	**Cluster 0** (Healthiest Skin)	**Cluster 1** (Acne-Prone & City Lifestyle)	**Cluster 2** (Pigmentation & Sun Exposure)	**Cluster 3** (Dry & Sensitive Skin)
Dietary Habits	Generally healthy/restricted diet reported often (mean 2.27; second-highest).	Less frequent healthy/restricted diet on average (mean 2.07).	Most frequent healthy/restricted diet among clusters (mean 2.37; highest).	Least frequent healthy/restricted diet among clusters (mean 2.04; lowest).
Exercise	Moderate weekly activity (mean 1.69; second-highest).	Lowest activity level on average (mean 1.11; lowest).	Intermediate activity (mean 1.37).	Highest activity level on average (mean 1.82; highest).
City Living	Lowest city-living prevalence (mean 0.24 $\approx$ 24% "Yes").	Highest city-living prevalence (mean 0.35 $\approx$ 35% "Yes").	Highest city-living prevalence (mean 0.35 $\approx$ 35% "Yes").	Moderately high city-living prevalence (mean 0.31 $\approx$ 31% "Yes").
Sunbathing	Moderate sun-exposure frequency (mean 0.98); similar to Cluster 2 and close to Cluster 3.	Lower sun-exposure frequency than other clusters (mean 0.75; lowest).	Moderate sun-exposure frequency (mean 0.97); similar to Cluster 0.	Slightly highest sun-exposure frequency, but differences are small (mean 1.01).
Scrub Usage	Lowest prevalence of granular scrub $>$1/week (mean 0.37 $\approx$ 37% "Yes").	High prevalence of scrub usage (mean 0.78 $\approx$ 78% "Yes").	Highest prevalence of scrub usage (mean 0.84 $\approx$ 84% "Yes").	Moderate prevalence of scrub usage (mean 0.46 $\approx$ 46% "Yes").
Menopause	Lower menopause prevalence (18.7% "Yes").	Lowest menopause prevalence (12.5% "Yes").	Intermediate menopause prevalence (26.6% "Yes").	Highest menopause prevalence (64.6% "Yes").

SUPPLEMENTARY 8: ROBUSTNESS CHECK: LEAKAGE-FREE K-FOLD CROSS-VALIDATION

As an additional robustness check, a leakage-free K-fold protocol was evaluated in which K-modes clustering, cluster-label assignment, and supervised prediction were performed within each fold. Specifically, K-modes was fit on the training partition only, and held-out samples were labeled using training-derived prototypes before classifier evaluation on the held-out fold. Using five folds, this procedure achieved accuracy 0.968 ± 0.007 with macro-precision 0.969 ± 0.008, macro-recall 0.966 ± 0.008, and macro-F1 0.967 ± 0.008. The leading predictors identified by Random Forest impurity-based importance were consistent with the fold-aggregated importance trends reported under nested cross-validation, with prominent contributions from lifestyle and exposure variables alongside matrix remodeling and oxidative stress genes. These results support the stability of the fold-wise clustering-to-prediction pipeline.

A. Leakage-free 5-fold cross-validation performance (mean ± std).

**Accuracy**	**Macro-Prec.**	**Macro-Rec.**	**Macro-F1**
0.968 ± 0.007	0.969 ± 0.008	0.966 ± 0.008	0.967 ± 0.008

B. Top predictors from leakage-free 5-fold evaluation using Random Forest impurity-based importance

**Feature**	**Importance**
Camouflage Imperfections	0.0766
Sleeping Habits	0.0765
Dietary_Habits	0.0731
Stress_Level	0.0702
Exercise	0.0649
MMP-1	0.0609
AQP3	0.0555
GPX	0.0507
MMP-3	0.0488
SOD2	0.0477
Low_H2O	0.0455
Sunbathing	0.0446
Living Outdoors	0.0404
Is_Winter	0.0352
City_Living	0.0332

Supplementary 9: Abbreviations and Acronyms

This table below includes key acronyms used throughout the manuscript, particularly those related to feature selection, machine learning models, sensor data, and methodology techniques. Defining these abbreviations will assist readers in understanding the technical terminology used in the study.

**Abbreviation**	**Definition**
AI	Artificial Intelligence
ML	Machine Learning
CNN	Convolutional Neural Network
XAI	Explainable Artificial Intelligence
CV	Cross-Validation
SHAP	SHapley Additive exPlanations
SNP	Single Nucleotide Polymorphism
FLG	Filaggrin (gene related to skin barrier function)
AQP3	Aquaporin 3 (gene related to skin hydration)
MMP-1	Matrix Metalloproteinase 1 (gene involved in collagen degradation)
MMP-3	Matrix Metalloproteinase 3 (gene involved in collagen degradation)
SOD2	Superoxide Dismutase 2 (gene involved in oxidative stress response)
GPX	Glutathione Peroxidase (gene involved in oxidative stress response)
ANOVA	Analysis of Variance
NMI	Normalized Mutual Information
SPF	Sun Protection Factor
AC	Air Conditioning
RF	Random Forest
PCA	Principal Component Analysis
t-SNE	t-Distributed Stochastic Neighbor Embedding
K-Modes	K-Modes Clustering Algorithm (for categorical data)
CAM	Camouflage Imperfections (feature related to makeup usage)
Is_Winter	Binary variable indicating whether it is winter
Fly_AC_YN	Binary variable indicating frequent air conditioning or flying
Low_H2O	Low water intake ($<$1.5L/day)
